# Metabolism-Related Gene TXNRD1 Regulates Inflammation and Oxidative Stress Induced by Cigarette Smoke through the Nrf2/HO-1 Pathway in the Small Airway Epithelium

**DOI:** 10.1155/2022/7067623

**Published:** 2022-12-19

**Authors:** Qian Huang, Maocuo Peng, Yiya Gu, Jixing Wu, Yuan Zhan, Zhesong Deng, Shanshan Chen, Ruonan Yang, Jinkun Chen, Jungang Xie

**Affiliations:** ^1^Department of Respiratory and Critical Care Medicine, National Clinical Research Center of Respiratory Disease, Key Laboratory of Pulmonary Diseases of Health Ministry, Tongji Hospital, Tongji Medical College, Huazhong University of Science and Technology, Wuhan, Hubei 430030, China; ^2^Department of Science, Western University, 1151 Richmond Street, London, Ontario, Canada N6A 3K7

## Abstract

Chronic obstructive pulmonary disease (COPD), a small airway disease, is regarded as a metabolic disorder. To further uncover the metabolic profile of COPD patients, it is necessary to identify metabolism-related differential genes in small airway epithelium (SAE) of COPD. Metabolism-related differential genes in SAE between COPD patients and nonsmokers were screened from GSE128708 and GSE20257 datasets. KEGG, GO, and PPI analyses were performed to evaluate the pathway enrichment, term enrichment, and protein interaction of candidate metabolism-related differential genes, respectively. RT-PCR was used to verify the mRNA expression of the top ten differential genes. Western blotting was used to evaluate the protein expression of TXNRD1. TXNRD1 inhibitor auranofin (AUR) was used to assess the impact of TXNRD1 on oxidative stress and inflammation induced by cigarette smoke extraction (CSE). Twenty-four metabolism-related differential genes were selected. ALDH3A1, AKR1C3, CYP1A1, AKC1C1, CPY1B1, and TXNRD1 in the top ten genes were significantly upregulated after CSE simulation for 24 h in human bronchial epithelial (16HBE) cells. Among them, CYP1A1 and TXNRD1 also have a significant upregulation in primary SAE after simulation of CSE for 24 h. The overexpression of protein TXNRD1 has also been detected in 16HBE cells, primary SAE stimulated with CSE, and mouse lung exposed to cigarette smoke (CS). Additionally, inhibition of TXNRD1 with 0.1 *μ*M AUR alleviated the expression of IL-6 and reactive oxygen species (ROS) induced by CSE by activating the Nrf2/HO-1 pathway in 16HBE cells. This study identified twenty-four metabolism-related differential genes associated with COPD. TXNRD1 might participate in the oxidative stress and inflammation induced by CS by regulating the activation of the Nrf2/HO-1 pathway.

## 1. Introduction

Chronic obstructive pulmonary disease (COPD) is a small airway inflammatory disease with irreversible airflow restriction [1]. With high mortality and morbidity, it has caused a heavy economic and social burden worldwide [1]. Currently, the pathogenesis of COPD is complicated and not fully elucidated. Smoking is a major risk factor for COPD, and oxidative stress and inflammation induced by cigarette smoke (CS) remain a prominent contributor to COPD pathogenesis [2].

Cell metabolism, which primarily contains amino metabolism, glucose metabolism, and lipid metabolism, plays an important role in cell physiological function [3]. Disturbances in metabolic patterns can be regulated by abnormally expressed metabolism-related genes in diseases [4]. Metabolic disturbance contributed to numerous diseases including COPD, which is associated with smoking status [5–8]. Abnormalities of metabolism-related genes have been reported to contribute to the development of numerous diseases by regulating oxidation stress, inflammation, and apoptosis [9–11]. In our previous study, we found a disorder of lung cells in male patients with COPD [12]. Considering the airway epithelium as the first line of defense after stimulation of smoking [13], we hypothesized abnormal expression of metabolism-related genes in small airway epithelium (SAE) of COPD patients. However, the aberrant expression of metabolism-related genes in SAE and their underlying mechanism in COPD are poorly understood.

In this study, we aimed to calculate the metabolism-related genes participated in COPD by reanalyzing two expression profiles of SAE. Thereafter, 24 metabolism-related differential genes were discovered. Among them, the mRNA overexpression of two genes, CYP1A1 and TXNRD1, was confirmed in 16HBE cells and primary SAE after cigarette smoke extract (CSE) stimulation. Furthermore, we also explored the protein expression of TXNRD1 in the lungs of mice exposed to CS and of COPD patients. To better understand the role of TXNRD1, an investigation using the TXNRD1 inhibitor auranofin (AUR) has been conducted.

## 2. Methods

### 2.1. Data Collection

Two mRNA expression profiles of primary small airway epithelium (SAE), GSE128708 and GSE20257, were collected from the Gene Expression Omnibus (GEO) database (http://www.ncbi.nlm.nih.gov/geo/). Differentially expressed genes (DEGs) in SAE between COPD patients and nonsmokers were analyzed by GEO2R.

Amino metabolism-related and glucose metabolism-related genes were obtained from GSEA (https://www.gsea-msigdb.org/gsea/index.jsp). Respectively, 372 and 324 amino metabolism-related and glucose metabolism-related genes were attained. Lipid metabolism-related genes were collected from the Kyoto Encyclopedia of Genes and Genomes (KEGG) website (http://www.kegg.jp/blastkoala/) and the Molecular Signatures Database (MSigDB) website (https://www.gseamsigdb.org/gsea/msigdb/index.jsp) [14]. 1045 lipid metabolism-related genes were finally included in analysis (Supplementary table 1.1-1.3).

### 2.2. Reagents and Antibodies

Cigarette smoke extraction (CSE) was prepared as previously described [15]. Antibodies against *β*-actin (anti-*β*-actin, 66009-1-Ig), TXNRD1 (anti-TXNRD1, 11117-1-AP), and Nrf2 (anti-Nrf2, 16396-1-ap) were purchased from Proteintech. HO-1 (anti-HO-1, GB11845) was purchased from Servicebio. Auranofin (Ridaura, SKF-39162), the inhibitor of thioredoxin reductase (TXNRD1), was purchased from Selleck.cn.

### 2.3. Cell Culture and Stimulation

The human airway epithelial 16HBE cell line was purchased and cultured as previously described [16]. The cells were incubated at 37°C with 5% CO_2_. For experimentation, 16HBE cells were grown in 12-well plates until 70–80% confluence and then exposed to CSE with different concentrations for 24 hours.

The primary small airway epithelium (SAE, 10th-12th generation bronchi) was collected by fiberoptic bronchoscopy from one healthy nonsmoker (male, 50 years old). Ten freshly brushed cells were washed with cell culture medium (DMEM, Lonza, Walkersville, MD) with 10% fetal bovine serum (FBS, Gibco) and penicillin-streptomycin (1 : 100). The collected cells were then centrifuged at 1000 rpm for 5 minutes and suspended with bronchial epithelial basal medium (BEBM) (Lonza, Walkersville, MD). Suspended cells (passage zero) were then inoculated into cell culture dishes, and fluid was changed every two days. Cell passage was performed when the degree of cell fusion reached 90 to 100%. Cells in passage two were grown in 12-well plates until 70–80% confluence and then exposed to CSE with different concentrations for 24 hours.

This study was authorized by the ethics committee of the Tongji Hospital, Huazhong University of Science and Technology in Wuhan, China. Each participant signed the written consent form prior to participation.

### 2.4. Cell Viability Assay

The viability of 16HBE cells treated with different concentrations of auranofin (AUR) was gauged using a CCK8 assay kit (CCK8; Promoter Biotechnology, Wuhan, China) according to the manufacturer's instructions. OD at 450 nm was determined with a microplate reader (Multiskan MK3; Thermo Fisher Scientific, Waltham, MA, USA).

### 2.5. Animal Model

10-12-week C57BL/6J mice were exposed to cigarette smoke in a chamber 3 hours daily for 6 months with Marlboro red cigarettes [17]. After six months of cigarette smoke exposure, anesthetized mice with 1% pentobarbital sodium 10 ml/kg body weight were sacrificed to collect lung tissue. All experimental procedures were approved by Huazhong University Animal Experiment Ethics Committee and were conducted in accordance with the animal experimentation guidelines of Huazhong University.

### 2.6. Immunofluorescence Analysis

Human lung and mouse left lung tissues were collected and placed in fresh 4% neutral-buffered paraformaldehyde for 24 hours at room temperature, then embedded in paraffin, and subjected to the histological analysis as previously reported [16]. Immunofluorescence staining was performed on human lung and mouse lung sections with anti-TXNRD1 (1 : 200).

### 2.7. ELISA

Cell culture supernatants were collected and centrifuged at 980 rpm for 15 minutes at 4°C and then stored at -80°C until use as the previous report [16]. ELISA kits for human IL8 (DY208) and human IL6 (DY206) were purchased from R&D systems. ELISA assay was carried out according to the instructions.

### 2.8. Reactive Oxygen Species (ROS) Detection

After treatment with CSE or/and AUR, ROS detection was conducted using the ROS assay kit (Servicebio, G1706-100T). Briefly, CSE-exposed 16HBE cells were washed twice with PBS and then incubated with DCFH-DA (diluted with RPMI1640, a dilution of 1 : 1000). After 30 minutes of opaque incubation in 37°C, cells were washed twice with PBS. Next, cells were directly observed under a fluorescence microscope or digested with trypsin and then analyzed by flow cytometry.

### 2.9. Western Blotting

Western blotting was performed as previously described. Total protein from mouse right lung tissue, small airway epithelium (SAE), or 16HBE cells was extracted by RIPA lysis buffer containing a protease inhibitor cocktail and phosphatase inhibitors (Roche, Mannheim, Germany). The proteins were separated by 10% SDS-PAGE and then transferred to polyvinylidene fluoride (PVDF) membranes (Millipore, Germany). The membranes were blocked for 1-2 hours in 5% milk melted in Tris-buffered saline containing 0.05% Tween 20 (TBST) and then incubated with the primary antibody (anti-*β*-actin, 1 : 4000; anti-TXNRD1, 1 : 2000; anti-Nrf2,1 : 2000; and anti-HO-1, 1 : 2000).

### 2.10. Quantitative RT-PCR Analysis

16HBE cells and primary SAE RNA were extracted by the TRIzol reagent method (Invitrogen). Total RNA was used for first-strand cDNA synthesis with M-MLV reverse transcriptase (Promega, Madison, WI). qRT-PCR was performed utilizing SYBR Green Master Mix (Takara, Otsu, Shiga, Japan) on the iCycler iQ system (Bio-Rad). PCR conditions included initial denaturation at 95°C for 5 minutes, 95°C for 45 seconds, and 60°C for 1 minute for 45 cycles. Gene expression levels were normalized to *β*-actin. The primers for genes are shown in Supplementary table 2.

### 2.11. Statistical Analysis

GO and KEGG pathway enrichment analyses were performed at http://www.bioinformatics.com.cn/; PPI analysis of differentially metabolism-related genes was analyzed using the STRING database (https://string-db.org) and Cytoscape software (version 3.8.1).

Data from *n* independent experiment were presented as means ± SEM. Normality analysis was performed via the Shapiro-Wilk test. Differences were evaluated using unpaired Student's *t* test between two groups before any testing. One-way ANOVA was performed followed by the Bonferroni post hoc test for comparisons between >2 groups. The nonnormal distributed data were analyzed using nonparametric testing (Mann-Whitney *U* test for two groups and Kruskal-Wallis *H* test for >2 groups). *P* values less than 0.05 were considered statistically significant. Statistical analysis was performed using GraphPad Prism 8.0.1 (GraphPad Software Inc., San Diego, CA).

## 3. Results

### 3.1. Metabolism-Related Differential Genes in the Small Airway Epithelium of COPD Patients versus Nonsmokers

**Figure 1:**
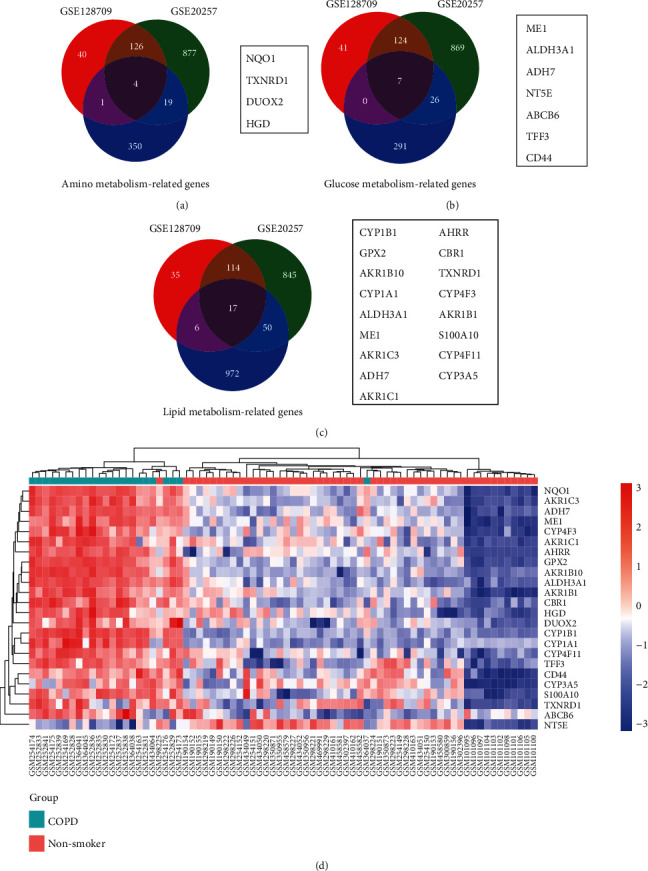
Metabolism-related genes associated with COPD in small airway epithelium. Venn diagrams showed the amino metabolism-related genes (a), glucose metabolism-related genes (b), and lipid metabolism-related genes (c). The heatmap of 24 metabolism-related differential genes (d).

The mRNA expression profile of small airway epithelium (SAE), GSE128708 [18, 19], was initially used to identify metabolism-related differential genes between COPD patients and nonsmokers. Thereafter, 35 metabolism-related differential genes were calculated in GSE128708. A total of 5, 7, and 23 genes belonged to amino, glucose, and lipid metabolism-related genes, respectively (Figures 1(a)–1(c)). To further validate the repeatability of our findings, profile GSE20257 [20] was also included in analysis. 171 differential genes associated with COPD were discovered in GSE20257. Among them, 4 of 5 amino metabolism-related genes including NQO1, TXNRD1, DUOX2, and HGD, 7 of 7 glucose metabolism-related genes including ME1, ALDH3A1, ADH7, NT5E, ABCB6, TFF3, and CD44, and 17 of 23 lipid metabolism-related genes including CPY1B1, GPX2, AKR1B10, CYP1A1, ALDH3A1, ME1, AKR1C3, ADH7, AKC1C1, AHRR, CBR1, TXNRD1, CYP4F3, AKR1B1, S100A10, CYP4F11, and CYP3A5 in GSE128708 were also identified in GSE20257 (Figures 1(a)–1(c)). Among them, TXNRD1 was involved in both lipid metabolism and amino metabolism, and genes ALDH3A1, ADH7, and ME1 participated in both lipid metabolism and glucose metabolism. Finally, a total of 24 metabolism-related differential genes in small airway epithelium of COPD patients versus nonsmokers were presented in a heatmap (Figure 1(d)).

### 3.2. KEGG, GO Pathway Enrichment, and PPI Network Analyses of the Differentially Expressed Metabolism-Related Genes

**Figure 2:**
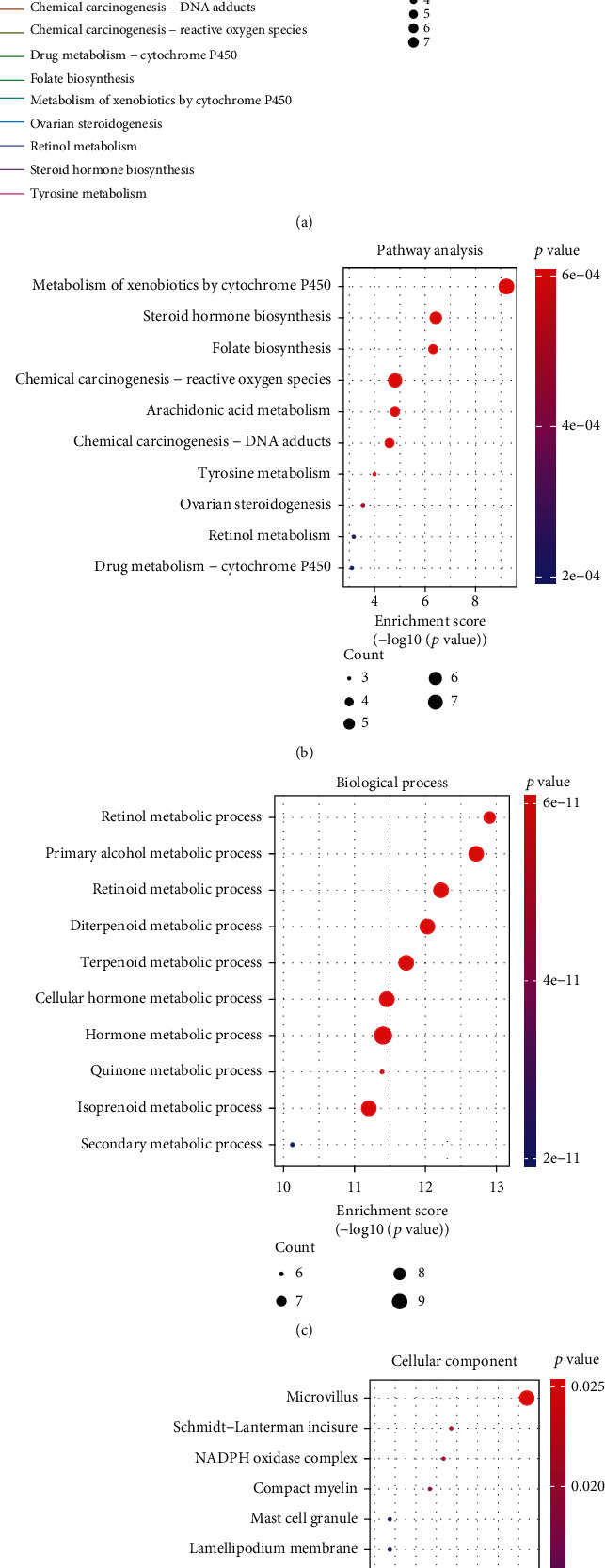
The KEGG pathways (a, b) and Gene Ontology (GO) enrichment terms (c, d) of the 24 metabolism-related differential genes associated with COPD in small airway epithelium.

KEGG and GO enrichment analyses were conducted to investigate the potential biological functions of 24 metabolism-related differential genes. KEGG pathway analysis showed that the top three pathways of the 24 metabolism-related differential genes were primarily involved in the metabolism of xenobiotics by cytochrome P450, chemical carcinogenesis-reactive oxygen species, and steroid hormone biosynthesis (Figures 2(a) and 2(b) and Supplementary table 3). GO enrichment analysis revealed that the most significant GO-enriched terms were related to metabolism (Figures 2(c) and 2(d) and Supplementary table 4).

**Figure 3:**
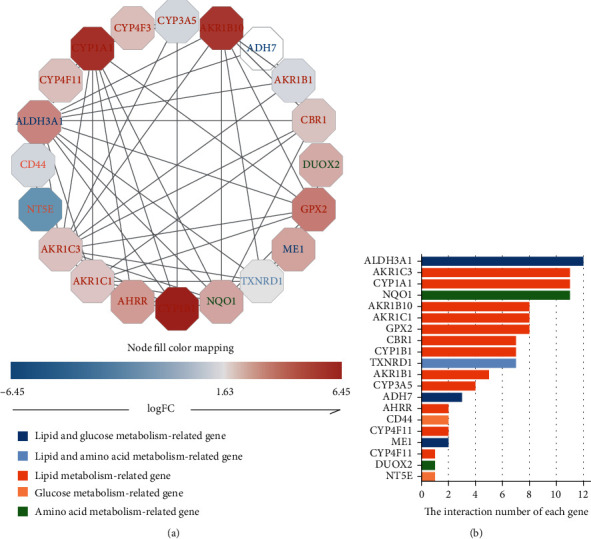
Protein-protein interaction (PPI) analysis of 24 metabolism-related differential genes (a) and the interaction number of each differentially expressed metabolism-related gene (b).

PPI analysis was implemented to determine the interactions among differentially expressed metabolism-related genes. There was a significant interaction between 24 metabolism-related genes (Figure 3(a)), and we also showed the interaction number of each gene (Figure 3(b)).

### 3.3. Validation of Metabolism-Related Differential Genes in Human Bronchial Epithelial Cells and Primary SAE

**Figure 4:**
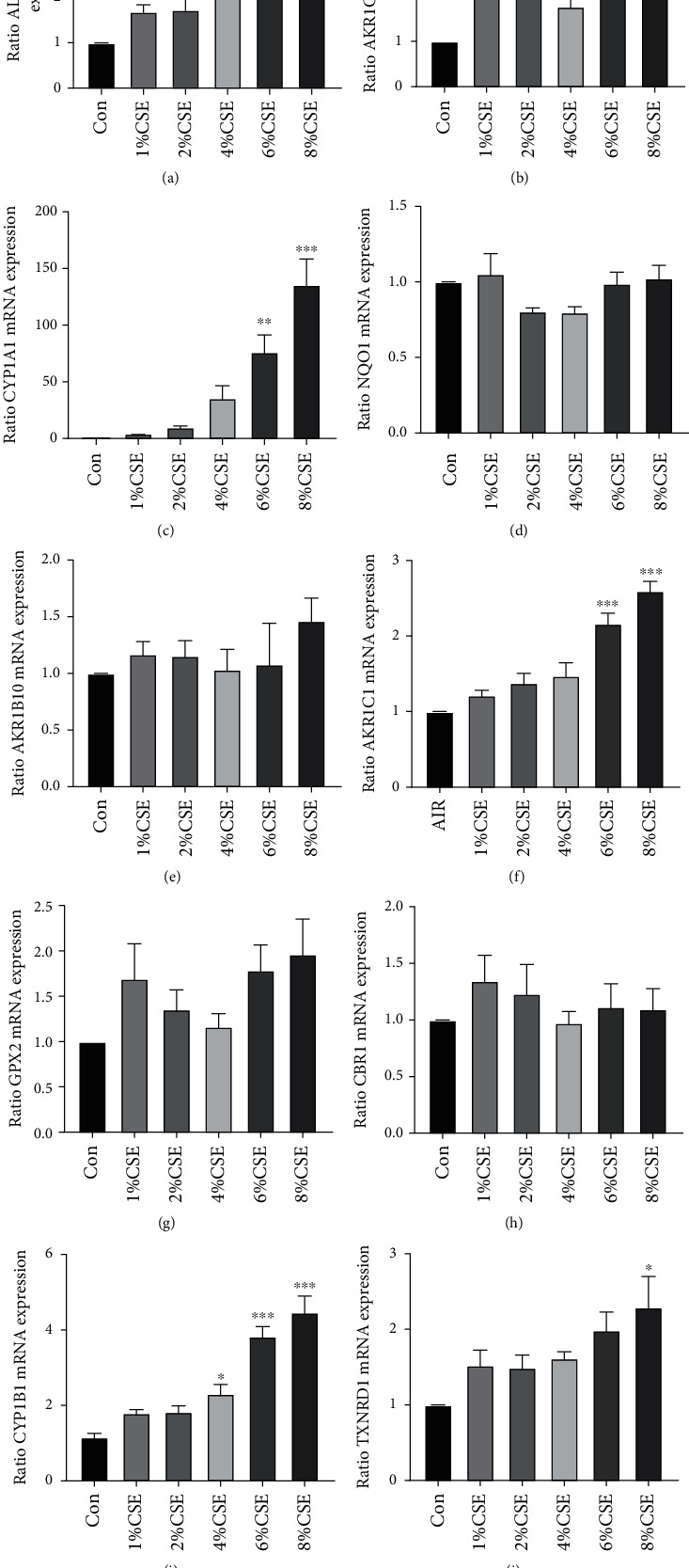
The validation of mRNA expression of top 10 metabolism-related differential genes in 16HBE cells. RT-PCR to calculate the expression of ALDH3A1 (a), AKR1C3 (b), CYP1A1 (c), NQO1 (d), AKR1B10 (e), AKC1C1 (f), GPX2 (g), CBR1 (h), CPY1B1 (i), and TXNRD1 (j).

**Figure 5:**
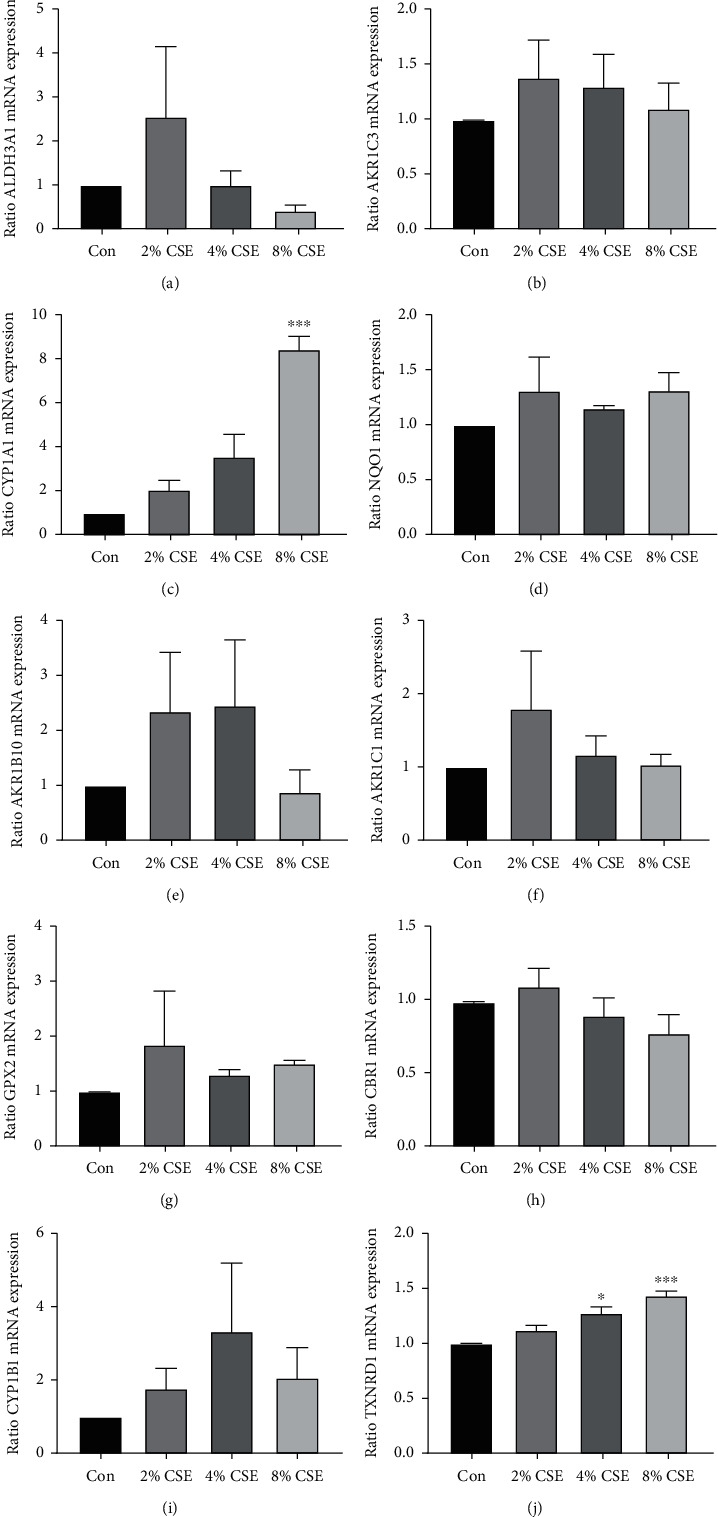
The validation of mRNA expression of top 10 metabolism-related differential genes in small airway epithelium. RT-PCR to calculate the expression of ALDH3A1 (a), AKR1C3 (b), CYP1A1 (c), NQO1 (d), AKR1B10 (e), AKC1C1 (f), GPX2 (g), CBR1 (h), CPY1B1 (i), and TXNRD1 (j).

To further filter out the most potential metabolism-related genes, the expression of mRNA of the top 10 of 24 genes was determined by RT-PCR in human bronchial epithelial (16HBE) cells and SAE. Six of ten genes including ALDH3A1, AKR1C3, CYP1A1, AKC1C1, CPY1B1, and TXNRD1 had a significant upregulation after simulation of cigarette smoke extraction (CSE) in 16HBE cells (Figures 4(a)–4(j)). Among them, CYP1A1 and TXNRD1 also showed a significant increase in primary SAE stimulated with CSE (Figures 5(a)–5(j)).

### 3.4. Protein Expression of TXNRD1 in 16HBE Cells, Primary SAE Stimulated with CSE, and Mouse Lungs Exposed to Cigarette Smoke

**Figure 6:**
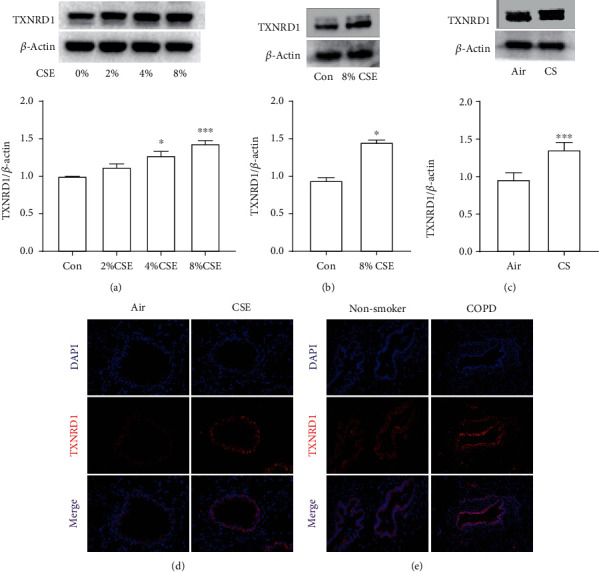
Western blotting to analyze the protein expression of TXNRD1. The expression of TXNRD1 in 16 HBE cells (a), small airway epithelium (b), and mouse lung (c).

CYP1A1 and TXNRD1 are two promising metabolism-related genes in our study. CYP1A1 was discovered to play a role in COPD by regulating oxidative stress. However, the function of TXNRD1 in COPD is rarely known. Therefore, we next explore the protein expression of TXNRD1. A significant overexpression of TXNRD1 has been calculated both in 16HBE cells, in primary SAE stimulated with CSE, and in mouse lung exposed to cigarette smoke (CS) (Figures 6(a)–6(e)).

### 3.5. Inhibition of TXNRD1 Reduced Inflammation and Oxidative Stress Induced by Cigarette Smoke Extract by Activating the Nrf2/HO-1 Pathway

Thioredoxin reductase 1 (TXNRD1) has been reported as a regulator of Nrf2 [21]. Nrf2 is a promising therapeutic target in COPD, which can regulate oxidative stress and inflammation by activating antioxidant response element-regulated antioxidant and cytoprotective genes [22, 23]. Moreover, inhibition of TXNRD1 with its inhibitor auranofin (AUR) can alleviate inflammatory reaction [24]. Heme oxygenase-1 (HO-1) is a downstream gene of Nrf2 [25], which can also be induced by TXNRD1 inhibition [26]. Hence, we hypothesized that TXNRD1 may participate in the development of COPD by regulating inflammation and oxidative stress and activating the Nrf2/HO-1 pathway.

**Figure 7:**
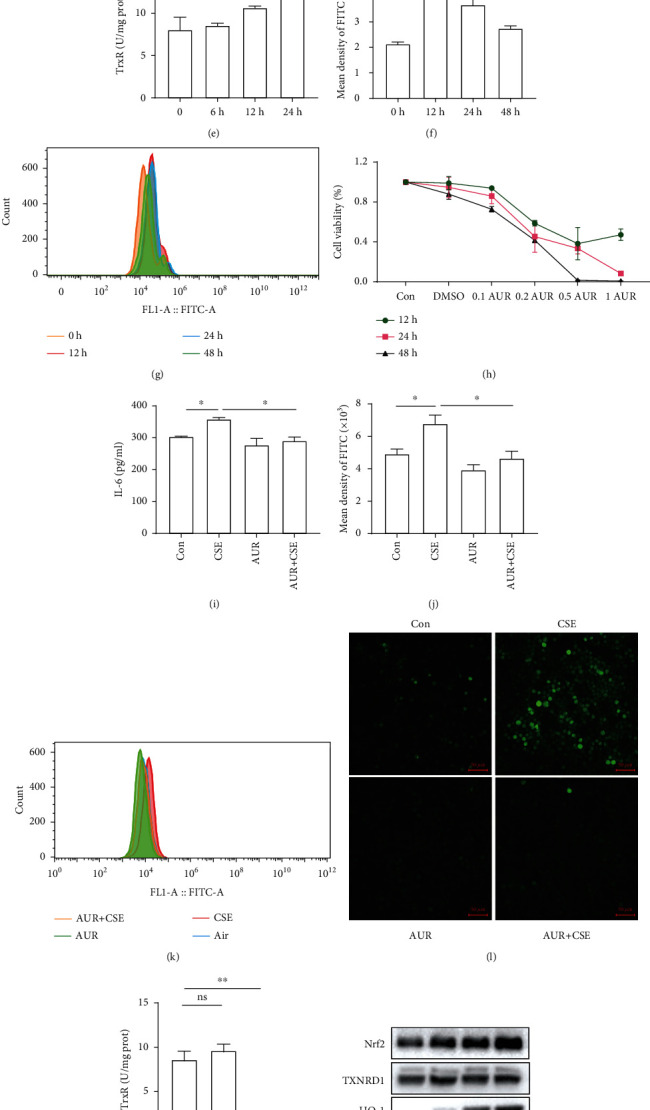
Auranofin (AUR) reduced IL-6 and reactive oxygen species (ROS) expression induced by cigarette smoke extract by activating the Nrf2/HO-1 pathway. Western blotting to analyze the protein expression of TXNRD1, Nrf2, and HO-1 after stimulating different times with 8% CSE in 16HBE cells (a–d). The effect of CSE on the activity of TXNRD1 (e) and the generation of reactive oxygen species (f, g). CCK8 to evaluate the impact of AUR on 16HBE cell viability (h). ELISA to detect the IL-6 level in 16HBE cells after simulation of CSE with or without 0.1 *μ*M AUR (i). The influence of AUR on ROS induced by CSE (j–l). The effect of AUR on the activity of TXNRD1 (m). Western blotting to analyze the expression of TXNRD1, Nrf2, and HO-1 in 16HBE cells after simulation of CSE with or without 0.1 *μ*M AUR (n–q).

We first determined the protein expression of TXNRD1, Nrf2, and HO-1 after stimulating different times with 8% CSE in HBE cells. CSE simulation increased the level of TXNRD1, Nrf2, and HO-1. There were significant upregulations of Nrf2 and HO-1 in 12 h after 8% CSE exposure, which gradually subsided. However, there was an increase of TXNRD1 at 24 h, which became more significant at 48 h (Figures 7(a)–7(d)). Additionally, the activity of TXNRD1 and the generation of reactive oxygen species (ROS) have also been examined. 8% CSE exposure enhanced the activity of TXNRD1 at 48 h (Figure 7(e)). CSE exposure significantly increased intracellular ROS at both 12 h, 24 h, and 48 h (Figure 7(f)).

Subsequently, the impact of inhibitor AUR on cell viability was evaluated. 0.1 *μ*M AUR which had no significant influence in 16 HBE cell viabilities was chosen to apply to the next study (Figure 7(h)). In our study, we found that CSE can induce increased IL-6 and ROS expression. Further, 0.1 *μ*M AUR can reduce the increased IL-6 and ROS induced by CSE (Figures 7(g)–7(l)). Simultaneously, 0.1 *μ*M AUR can also considerably inhibit the activity of TXNRD1 and improved the activation of the Nrf2/HO-1 pathway without affecting TXNRD1 expression (Figures 7(m)–7(q)).

## 4. Discussion

In this study, we performed an analysis on the metabolism-related genes associated with small airway epithelium (SAE) of COPD. A total of 24 differential genes including 4 amino metabolism-related genes, 7 glucose metabolism-related genes, and 17 lipid metabolism-related genes were identified. mRNA expression of the top 10 genes was verified in 16HBE cells and primary SAE. In 16 HBE cells, the expression of the following genes was confirmed: ALDH3A1, AKR1C3, CYP1A1, AKC1C1, CPY1B1, and TXNRD1. The overexpression of CPY1A1 and TXNRD1 was also validated in SAE. Thereafter, it was confirmed that TXNRD1 protein overexpression occurred in the lungs of patients with COPD, mice exposed to cigarette smoke (CS), 16 HBE cells, and SAE treated with cigarette smoke extraction (CSE). Furthermore, inhibition of TXNRD1 with auranofin (AUR) mitigated the expression of IL-6 and ROS induced by CSE by activating the Nrf2/HO-1 pathway.

Metabolic dysfunctions of major metabolic pathways result in respiratory diseases by regulating mitochondrial dysfunction and oxidative stress, cellular senescence, inflammation, and aberrant T cell immune response [3]. Metabolic abnormalities are also a striking feature of COPD and contribute to the development of COPD [27]. Expectedly, the identification of metabolism-related genes to highlight the abnormal metabolic profile could benefit exploring new therapy targets for COPD [28].

Smoking is a major risk for COPD, which can change cell metabolism [7]. Airway epithelium is the first defense after exposure of CS [29]. Our study screened out 24 differential metabolism-related genes in SAE of COPD patients versus nonsmokers. Notably, these genes were mainly gathered in the pathway associated in oxidation stress. Oxidation stress plays a crucial role in COPD. In our study, the upregulated CYP1A1 and TXNRD1 have been verified both in 16 HBE cells and in SAE exposed to CSE. CYP1A1 metabolizes multiple exogenous and endogenous substrates, which is mainly controlled by the aryl hydrocarbon receptor (AHR) and has been found to be involved in lung disease including COPD by regulating inflammation and oxidative stress [30–32]. However, the function of thioredoxin reductase-1 (TXNRD1) in COPD is rarely known.

In this study, we found a significant protein overexpression of TXNRD1 in 16HBE cells, human primary SAE stimulated with CSE, and mouse lungs exposed to CS. TXNRD1 is an NADPH-dependent selenocysteine-containing oxidoreductase that catalyzes the reduction of oxidized thioredoxin-1 [26]. Regulating cellular phenotypes, cellular growth, and responses to stimuli [33], TXNRD1 is related to various cancer diseases and is also a popular target for cancer [34]. In addition, TXNRD1 inhibition has been reported to ameliorate LPS-induced inflammation [24]. TXNRD1 inhibitor AUR has the function of anti-inflammation and antioxidation [35]. Our study also found that TXNRD1 inhibition can alleviate the secretion of IL-6 and ROS induced by CSE in SAE, which can be a promising therapy target of COPD. AUR is an FDA-approved drug to treat rheumatoid arthritis (RA). Mechanically, AUR protected against oxidation damage by direct and indirect control of enzyme systems involved in the production or transformation of ROS [36, 37]. Anyhow, it is worth noting that TXNRD1 is a basal antioxidant enzyme and plays roles in antioxidant defense. Inhibition of TXNRD1 with AUR induced the release of ROS in cancer cells. We surmised that the contradictory impact of AUR on intracellular ROS might be associated with the cell types and disease states. Moreover, in our study, AUR can remarkably restrain the TXNRD1 activity and activated Nrf2/HO-1 signal. Whether the protective role of activating Nrf2/HO-1 offsets and exceeds the adverse role of TXNRD1 inhibition in ROS generation remains unclear.

TXNRD1 has been reported as a regulator of Nrf2 [21]. In COPD, Nrf2 is a promising therapeutic target because it can regulate oxidative stress and inflammation through activating response element-regulated antioxidant and cytoprotective genes [22, 23]. HO-1 is a downstream gene of Nrf2 [25]. The activation of Nrf2/HO-1 signaling regulates mitochondrial dysfunction, oxidation stress, and cellular senescence [38, 39]. Repression of Nrf2/HO-1 signaling aggravates emphysema and inflammation induced by elastase or CS [40, 41]. Although there is a conflicting expression of Nrf2 and HO-1 after CSE stimulation, this is dependent on the illness state or the duration of stimulation [42–44]. The protective effect of Nrf2/HO-1 signaling activation is adamantine [22, 39–41, 45]. We found that TXNRD1 inhibition can activate Nrf2/HO-1 signaling, and we conjecture that TXNRD1 regulates oxidative stress and inflammation through activating Nrf2/HO-1 signaling. The TXNRD1 inhibitor can be a promising therapy target of COPD.

There are some limitations in this study. First, more primary SAE from non-COPD and COPD patients needed to be collected and analyzed. Moreover, our study only partially revealed the effect of TXNRD1 inhibition with auranofin (AUR) on ROS generation and inflammation induced by CS. Experiments with subtraction and overexpression of TXNRD1 are also required to further verify TXNRD1 function in vivo and in vitro. Anyhow, the underlying mechanism of TXNRD1 and TXNRD1 inhibition on inflammation and oxidation stress is also needed to further explore, which is what we are investigating.

## 5. Conclusion

Our study determined metabolism-related genes associated with COPD. These genes primarily contribute to oxidation stress. We also validate the upregulation of TXNRD1 and its impact on oxidation stress and inflammation. Our study hints that inhibiting TXNRD1 activated Nrf2/HO-1 signaling and alleviated oxidation stress and inflammation, which can be a promising therapy target of COPD.

## Data Availability

All data used to support the findings of this study are included within the article. Raw data (GSE128709 and GSE20257) used to screen differential genes in small airway epithelium between COPD patients and nonsmokers were downloaded from the Gene Expression Omnibus (GEO) database (http://www.ncbi.nlm.nih.gov/geo/).

## Supplementary Materials

Supplementary Materials Supplementary table 1.1: the list of amino metabolism-related genes. Supplementary table 1.2: the list of glucose metabolism-related genes. Supplementary table 1.3: the list of lipid metabolism-related genes. Supplementary table 2: the primers for RT-qPCR in this study. Supplementary table 3: results of KEGG analysis of 24 differentially expressed metabolism-related genes. Supplementary table 4: results of GO pathway enrichment analysis of 24 differentially expressed metabolism-related genes.
