# Supplementary material for: Metabolism-Related Gene TXNRD1 Regulates Inflammation and Oxidative Stress Induced by Cigarette Smoke through the Nrf2/HO-1 Pathway in the Small Airway Epithelium
Source: Oxid Med Cell Longev. 2022 Dec 19;2022:7067623. doi: 10.1155/2022/7067623 (PMC9792251; doi:10.1155/2022/7067623)
Supplement: Supplementary Materials — Supplementary table 1.1: the list of amino metabolism-related genes. Supplementary table 1.2: the list of glucose metabolism-related genes. Supplementary table 1.3: the list of lipid metabolism-related genes. Supplementary table 2: the primers for RT-qPCR in this study. Supplementary table 3: results of KEGG analysis of 24 differentially expressed metabolism-related genes. Supplementary table 4: results of GO pathway enrichment analysis of 24 differentially expressed metabolism-related genes. [file 7067623.f1.zip › Supplementary table 2.docx]

**Table S2. The primers for RT-qPCR.**

| Name | Sequences |
| --- | --- |
| ALDH3A1 Forward | TGTTCTCCAGCAACGACAAGG |
| ALDH3A1 Reverse | AGGGCAGAGAGTGCAAGGT |
| AKR1C3 Forward | GTCATCCGTATTTCAACCGGAG |
| AKR1C3 Reverse | CCACCCATCGTTTGTCTCGTT |
| CYP1A1 Forward | ACATGCTGACCCTGGGAAAG |
| CYP1A1 Reverse | AACGTGCTTATCAGGACCTCA |
| NQO1 Forward | GAAGAGCACTGATCGTACTGGC |
| NQO1 Reverse | GGATACTGAAAGTTCGCAGGG |
| AKR1B10 Forward | GTGACACCAGCACGCATTG |
| AKR1B10 Reverse | GCATTGAAGGGATAGTCTTCCAA |
| AKR1C1 Forward | TCCAGTGTCTGTAAAGCCAGG |
| AKR1C1 Reverse | CCAGCAGTTTTCTCTGGTTGAA |
| GPX2 Forward | GAATGGGCAGAACGAGCATC |
| GPX2 Reverse | CCGGCCCTATGAGGAACTTC |
| CBR1 Forward | CAAGCTGAAGTGACGATGAAAAC |
| CBR1 Reverse | ATACGTTCACCACTCTCCCTT |
| CYP1B1 Forward | ACGTACCGGCCACTATCACT |
| CYP1B1 Reverse | ACGTACCGGCCACTATCACT |
| TXNRD1 Forward | ATGGGCAATTTATTGGTCCTCAC |
| TXNRD1 Reverse | CCCAAGTAACGTGGTCTTTCAC |
